# Immunoadsorption in dilated cardiomyopathy: the IASO-DCM trial

**DOI:** 10.1093/eurheartj/ehaf1055

**Published:** 2026-02-23

**Authors:** Stephan B Felix, Michael Böhm, Rüdiger C Braun-Dullaeus, Alida L P Caforio, Erland Erdmann, Ulrich Grabmaier, Stefan Groß, Karin Klingel, Fabian Knebel, Kristin Lehnert, Amir A Mahabadi, Veselin Mitrovic, Matthias Nauck, Georg Nickening, Michel Noutsias, Anja Sandek, Heinz-Peter Schultheiss, Christian Schulze, Heribert Schunkert, Petar M Seferovic, Karl Stangl, Alexander Staudt, Carsten Tschöpe, Rolf Wachter, Danilo Wegner, Karl Werdan, Finn Waagstein, Åke Hjalmarson, Marcus Dörr

**Affiliations:** Department of Internal Medicine B, University Medicine Greifswald, Ferdinand-Sauerbruch-Str., 17475 Greifswald, Germany; DZHK (German Centre for Cardiovascular Research), Partner Site North, Fleischmann-Str. 42, 17475 Greifswald, Germany; Clinic of Internal Medicine III and HOMICAREM (HOMburg Institute for CARdioRenalMetabolic Medicine), Saarland University, Homburg Saar, Germany; Division of Cardiology, Centre of Internal Medicine, University Hospital Magdeburg, Otto-von-Guericke University, Magdeburg, Germany; Unit of Cardiology, Department of Cardiac, Thoracic, Vascular Sciences and Public Health, University of Padova, Padova, Italy; Department of Internal Medicine III, University of Cologne, Cologne, Germany; Department of Medicine I, University Hospital, Ludwig Maximilian University of Munich, Munich, Germany; DZHK (German Centre for Cardiovascular Research), Partner Site Munich, Munich Heart Alliance, Munich, Germany; Department of Internal Medicine B, University Medicine Greifswald, Ferdinand-Sauerbruch-Str., 17475 Greifswald, Germany; DZHK (German Centre for Cardiovascular Research), Partner Site North, Fleischmann-Str. 42, 17475 Greifswald, Germany; Department of Cardiopathology, Institute for Pathology and Neuropathology, University Hospital Tübingen, Tübingen, Germany; Department of Cardiology, Angiology and Intensive Care Medicine, German Heart Center of the Charité (DHZC), Campus Charité Mitte, Berlin, Germany; DZHK (German Centre for Cardiovascular Research), Partner Site, Berlin, Germany; Department of Internal Medicine B, University Medicine Greifswald, Ferdinand-Sauerbruch-Str., 17475 Greifswald, Germany; DZHK (German Centre for Cardiovascular Research), Partner Site North, Fleischmann-Str. 42, 17475 Greifswald, Germany; Department of Cardiology and Vascular Medicine, West German Heart and Vascular Center Essen, University Hospital Essen, University of Duisburg-Essen, Essen, Germany; Department of Cardiology, Kerckhoff-Klinik, Bad Nauheim, Germany; DZHK (German Centre for Cardiovascular Research), Partner Site RheinMain, Frankfurt am Main, Germany; DZHK (German Centre for Cardiovascular Research), Partner Site North, Fleischmann-Str. 42, 17475 Greifswald, Germany; Institute of Clinical Chemistry and Laboratory Medicine, University Medicine Greifswald, Greifswald, Germany; Department of Internal Medicine II – Cardiology/Pneumology/Vascular Medicine, University Hospital Bonn, Bonn, Germany; Department of Internal Medicine I, Jena University Hospital – Friedrich Schiller University Jena, Jena, Germany; Clinic for Cardiology and Pneumology, University Medicine Göttingen, Göttingen, Germany; DZHK (German Centre for Cardiovascular Research), Partner Site Göttingen, Göttingen, Germany; Institute of Cardiac Diagnostics and Therapy, IKDT GmbH, Berlin, Germany; Department of Internal Medicine I, Jena University Hospital – Friedrich Schiller University Jena, Jena, Germany; DZHK (German Centre for Cardiovascular Research), Partner Site Munich, Munich Heart Alliance, Munich, Germany; Department of Cardiology, Deutsches Herzzentrum, TUM Universitätsklinikum, Technische Universität München, Munich, Germany; Belgrade University Medical Center, Belgrade University School of Medicine and Heart Failure Center, Belgrade, Serbia; Department of Cardiology, Angiology and Intensive Care Medicine, German Heart Center of the Charité (DHZC), Campus Charité Mitte, Berlin, Germany; DZHK (German Centre for Cardiovascular Research), Partner Site, Berlin, Germany; Department of Internal Medicine B, University Medicine Greifswald, Ferdinand-Sauerbruch-Str., 17475 Greifswald, Germany; Department of Cardiology and Angiology, Helios-Kliniken Schwerin, Schwerin, Germany; DZHK (German Centre for Cardiovascular Research), Partner Site, Berlin, Germany; Department of Cardiology, Angiology, and Intensive Medicine, German Heart Center of the Charité (DHZC), Campus Virchow, Charité Universitätsmedizin, Berlin, Germany; Berlin Institute of Health (BIH) at Charité, Berlin Brandenburg Center for Regenerative Therapies (BCRT), Berlin, Germany; Clinic for Cardiology and Pneumology, University Medicine Göttingen, Göttingen, Germany; DZHK (German Centre for Cardiovascular Research), Partner Site Göttingen, Göttingen, Germany; Department of Cardiology, University Hospital Leipzig, Leipzig, Germany; Department of Internal Medicine B, University Medicine Greifswald, Ferdinand-Sauerbruch-Str., 17475 Greifswald, Germany; DZHK (German Centre for Cardiovascular Research), Partner Site North, Fleischmann-Str. 42, 17475 Greifswald, Germany; Department of Internal Medicine III – Cardiology, Angiology and Internal Intensive Care Medicine, Mid-German Heart Center, University Hospital Halle (Saale), Halle, Germany; Wallenberg Laboratory, Sahlgrenska University Hospital, Göteborg, Sweden; Wallenberg Laboratory, Sahlgrenska University Hospital, Göteborg, Sweden; Department of Internal Medicine B, University Medicine Greifswald, Ferdinand-Sauerbruch-Str., 17475 Greifswald, Germany; DZHK (German Centre for Cardiovascular Research), Partner Site North, Fleischmann-Str. 42, 17475 Greifswald, Germany

**Keywords:** Dilated cardiomyopathy, Left ventricular ejection fraction, Immunoadsorption therapy

## Introduction

Dilated cardiomyopathy (DCM) is characterized by left ventricular (LV) dilatation and systolic dysfunction without abnormal loading conditions.^1–4^ Chronic heart failure (HF) treatments have improved outcomes, but 10-year event-free survival remains low at 42%.^3^

Genetic factors,^1–4^ inflammation, and cardiac-specific antibodies may drive disease progression.^5,6^ If relevant, removing cardiac antibodies may improve patient outcomes. Small studies have shown that removal of immunoglobulin G (IgG) antibodies by immunoadsorption (IA) improves LV ejection fraction (LVEF) and symptoms of patients with DCM.^6,7^ But randomized and blinded data are still lacking.

## Methods

### Study design

The ImmunoAdSorptiOn on cardiac function in patients with Dilated CardioMyopathy trial (IASO-DCM) was an investigator-initiated multicentre, double-blind, sham-controlled, randomized phase 2 study (registration number: NCT00558584).^8^ This trial evaluated the effects of IA and subsequent IgG substitution (IA/IgG) on LVEF after 6 months using contrast-enhanced echocardiography in patients with DCM.

Key inclusion criteria were DCM, LVEF < 40%, New York Heart Association (NYHA) classes II–IV, HF symptoms for 6 months to 7 years, and guideline-directed HF treatment for at least 6 months, with stable doses for a minimum of 2 months.^8^

In the IA/IgG group, immunoglobulins were removed by IA columns in five consecutive daily sessions, each processing two to three plasma volumes.^8^ Six hours after the final session, .5 g/kg of polyclonal IgG was infused intravenously to restore IgG levels and reduce infection risk. The control group underwent sham IA (plasmapheresis followed by reinfusion of the plasma without passing through adsorption columns) followed by an intravenous saline infusion.

All assessments were blinded. Contrast-enhanced echocardiography was performed at baseline and 6 months.^8^

Serum samples obtained at baseline were analysed for the presence of anti-heart and anti-endothelial cell antibodies by indirect immunofluorescence as described earlier.^9^ Endomyocardial biopsies were performed when clinically indicated. Myocardial inflammation was diagnosed as previously described.^10^ Nested polymerase chain reaction or reverse transcriptase polymerase chain reaction was performed to detect viruses.^10^

### Outcomes

All patients were included in the intention-to-treat analysis set. Statistical methods have been described previously.^8^

The primary endpoint was the change in LVEF from baseline to 6 months as determined by contrast echocardiography and evaluated by the core laboratory.^8^ The key secondary endpoint was a composite of all-cause mortality, cardiac resuscitation, hospitalization for HF, or cardiac surgery [heart transplantation, ventricular assist device (VAD) implantation, or biventricular pacing] within 24 months.^8^ Additional secondary endpoints included LV end-systolic and end-diastolic volume indices (LVESVI and LVEDVI), NYHA class, and Minnesota Living with HF Questionnaire (MLHFQ) scores.^8^

Safety endpoints included serious adverse events (SAEs) during the intervention period and at 1, 3, and 6 months, as well as a 6-month composite of all-cause death and hospitalization due to cardiovascular events or infections.^8^

## Results

### Patient characteristics

Between November 2008 and December 2018, 576 outpatients were screened and 83 outpatients were assigned to the IA/IgG group and 88 outpatients to the control group. An interim analysis conducted in July 2017, after 101 patients completed their 6-month evaluation, showed a 4% between-group difference in LVEF change (*P* = .008), falling between the predefined significance (*P* < .005) and futility (*P* > .22) boundaries. Due to slow enrolment, the sponsor decided to stop recruitment prematurely at the end of 2018.

**Figure 1:**
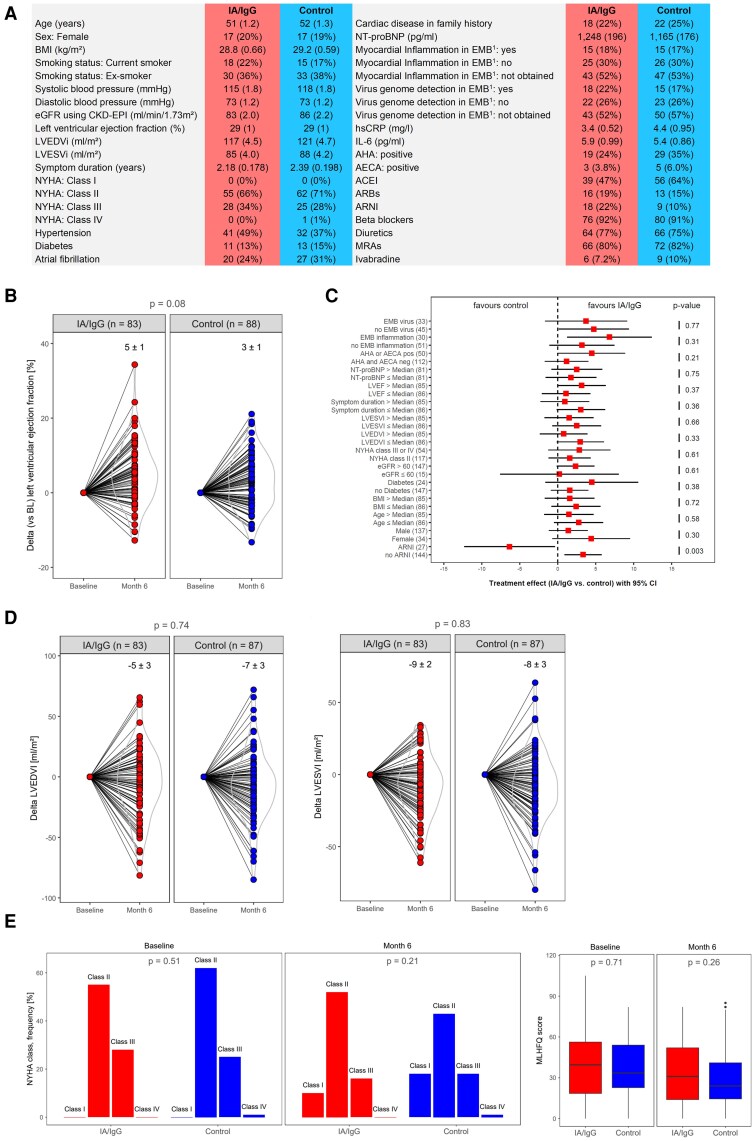
(*A*) Baseline characteristics of the patients and effects of IA/IgG treatment (IA/IgG) as compared with sham intervention (Control). Continuous parameters are given as mean ± SEM and categorical parameters as absolute numbers and percentages. EMB was not mandatory and was only performed for clinical reasons at discretion of the investigators prior to study inclusion. Viral genome Polymerase Chain Reaction revealed parvovirus B19 (IA/IgG *N* = 16; control *N* = 12), human herpes virus (IA/IgG *N* = 4; control *N* = 5), and Epstein–Barr virus (IA/IgG *N* = 2; control *N* = 1). (*B*) Connected point/violin plot of change of LVEF (delta %) expressed by the change from baseline to 6 months connected for each individual patient per treatment group. Numbers indicate mean ± SEM in the IA/IgG group and the control group. The numbers in brackets indicate the number of patients. Lines denote each individual patient per treatment group. The *P*-value indicates the statistical difference between the IA/IgG group and the control group. (*C*) Prespecified and *post hoc* subgroup analysis of the primary endpoint (change in LVEF from baseline to 6 months). Point estimates indicate the treatment effect of IA/IgG vs control for change in LVEF from baseline to 6 months with 95% CI. No adjustments for multiplicity were applied as those subgroup analyses were only considered exploratory hypothesis-generating analyses. The numbers in brackets indicate the number of patients. The *P*-values indicate differential treatment effects between the according subgroups (interaction: subgroup × treatment group). NT-proBNP: median = 576 pg/mL; LVEF: median = 29.1%; symptom duration: median = 1.56 years; ESVI: median = 76.7 mL/m^2^; EDVI: median = 111 mL/m^2^; BMI: median = 28.4 kg/m²; age: median = 53.4 years. (*D*) Change in left ventricular volumes. Connected point/violin plot of change of LVEDVI and LVESVI (delta mL/m^2^) expressed by the change from baseline to 6 months. Data presentation and statistical annotations are described in (B). (*E*) Change in functional status: NYHA classification and MLHFQ score in the IA/IgG group and the control group at baseline and after 6 months. The *P*-values indicate the statistical difference between the IA/IgG group and the control group. Median and interquartile range were used for MLHFQ score analysis. IA/IgG denotes treatment group. In this group, immunoglobulins were removed by immunoadsorption (IA) followed by intravenous immunoglobin G (IgG) infusion to restore IgG levels. Control denotes the control group. In this group, sham IA took place (plasmapheresis with subsequent reinfusion of the plasma without passing through adsorption columns) followed by an intravenous saline infusion instead of IgG infusion. AHA, anti-heart antibodies; AECA, anti-endothelial antibodies; BMI, body mass index; CKD-EPI, Chronic Kidney Disease Epidemiology Collaboration; eGFR, estimated glomerular filtration rate; EMB, endomyocardial biopsy; EMB virus, detection of virus genome in endomyocardial biopsies; EMB inflammation, detection of myocardial inflammation in endomyocardial biopsies; hs-CRP, high-sensitivity C-reactive protein; IL-6, interleukin 6; LVEF, left ventricular ejection fraction; LVEDVI, left ventricular end-diastolic volume divided by body surface area; LVESVI, left ventricular end-systolic volume divided by body surface area; MLHFQ score, Minnesota Living with HF Questionnaire score; NT-proBNP, N-terminal pro-B-type natriuretic peptide; NYHA class, New York Heart Association class; SEM, standard error of the mean; Symptom duration, symptom duration of HF. Medication: ACEI, angiotensin-converting enzyme inhibitor; ARBs, angiotensin II receptor blockers; ARNI, angiotensin receptor II blocker-neprilysin inhibitor sacubitril/valsartan; MRAs, mineralocorticoid receptor antagonists.IA/IgG denotes the treatment group.

Baseline clinical characteristics and HF medication (*Figure 1A*) were comparable between the groups, except for sacubitril/valsartan, which was used more frequently in the treatment group compared with the control group (*N* = 18 vs *N* = 9). Baseline blood samples were available from 162 patients and stored at −80°C for later analyses. Data from immunohistological (*N* = 81) and viral diagnostic (*N*= 78) analyses of endomyocardial biopsies were available for a subset of patients. These biopsies had been obtained for clinical reasons prior to study inclusion (mean 11.7 ± 1.75 months). Fifty patients were tested positive for cardiac antibodies.

### Efficacy outcomes

All patients tolerated IA/IgG well. Antibodies were effectively removed by IA. In the control group, plasma IgG decreased from 10.35 ± .32 to 8.9 ± .29 g/dL at Day 5. In the IA/IgG group, plasma IgG levels decreased from 10.48 ± .30 to .8 ± .18 g/dL at Day 5 after the last IA session before IgG substitution (*P* < .001 vs control).

After 6 months, LVEF increased from 29 ± 1 to 34 ± 1% in the IA/IgG group and from 29 ± 1 to 32 ± 1% in the control group. The increase in LVEF did not differ significantly (*P* = .08) between the IA/IgG group and the control group (*Figure 1B*). The neutral effect of IA/IgG on LVEF was consistent across all subgroups except one (*Figure 1C*). A *post hoc* analysis revealed a significant interaction in patients receiving sacubitril/valsartan, a medication newly introduced in the guidelines during the study period (*P* = .003).

### Secondary endpoints

The composite key secondary endpoint as assessed by time to first event occurred in 19 patients in the IA/IgG group and in 15 patients in the control group [hazard ratio (HR): 1.42, 95% confidence interval (CI): .72–2.80, *P* = .31]. No significant differences were observed in the individual components: all-cause death (HR: 1.06, 95% CI: .21–5.25), cardiac surgery (HR: 1.38, 95% CI: .48–3.99), cardiac resuscitation (0 vs 3 events), and hospitalization for HF (HR: 1.50, 95% CI: .71–3.16). SAEs were comparable (HR: 1.08, 95% CI: .47–2.5, *P* = .85), with no deaths recorded in either group at 6 months.

After 6 months, LVEDVI decreased from 117 ± 5 to 112 ± 5 mL/m^2^ in the IA/IgG group and from 120 ± 5 to 113 ± 5 mL/m^2^ in the control group. LVESVI decreased from 85 ± 4 to 76 ± 4 mL/m^2^ and from 87 ± 4 to 79 ± 4 mL/m^2^, respectively. The decrease in LV volumes was similar between groups (*Figure 1D*).

Functional status (NYHA class and MLHFQ score) was similar between groups at baseline and 6 months (*Figure 1E*).

## Discussion

This study showed that IA/IgG was safe and well tolerated in patients with DCM and HF but did not significantly improve LVEF or HF symptoms after 6 months compared with sham treatment. The key secondary endpoint was also similar. Thus, IA/IgG is not a general treatment option in DCM.

While the study was adequately powered overall, subgroup analyses—particularly those involving patients with myocardial inflammation, cardiac autoantibodies, or those taking sacubitril/valsartan—were limited by small sample sizes. These mechanistic aspects remain of interest and will be explored in future analyses to generate hypotheses.

## Data Availability

The data that support the findings of this study are available on request from the corresponding author (S.B.F.). Due to legal regulations, the data are not publicly available.

## Pre-registered Clinical Trial Number

Clinical trial registration: NCT00558584.
